# From CPAP Prescription to Long-Term Follow-Up: Care Pathway, Adherence, and Multidomain Treatment Outcomes in Obstructive Sleep Apnea

**DOI:** 10.3390/jcm15218296

**Published:** 2026-10-24

**Authors:** Giulia Sartori, Rosaria Caprino, Ernesto Crisafulli

**Affiliations:** Respiratory Medicine Unit, Department of Medicine, University of Verona and Azienda Ospedaliera Universitaria Integrata of Verona, Largo L.A. Scuro, 10, 37134 Verona, Italy

**Keywords:** obstructive sleep apnea, continuous positive airway pressure, CPAP adherence, long-term follow-up, care pathway, patient-reported outcomes, residual sleepiness

## Abstract

**Background/Objectives:** Long-term continuous positive airway pressure (CPAP) management involves both engagement with care and sustained treatment outcomes. We aimed to characterize long-term follow-up attendance, longitudinal adherence, and multidomain treatment outcomes in patients with obstructive sleep apnea (OSA). **Methods:** We studied 172 adults with OSA who entered a real-world CPAP care pathway. Long-term attendance was evaluated among patients with ≥30 months of potential follow-up from CPAP prescription, with 24- and 36-month thresholds examined in sensitivity analyses. Longitudinal adherence and multidomain outcomes were assessed among patients with available follow-up data. **Results:** Among 114 patients in the primary long-term follow-up risk set, 80 (70.2%) attended; attendance was similar using 24- and 36-month thresholds (68.5% and 72.8%). Older age was associated with greater attendance (odds ratio 1.58 per 10-year increase, 95% confidence interval 1.10–2.28). Among 94 patients with paired adherence data, adherence according to available device-recorded nights was 78.7% at first and 79.8% at long-term follow-up, although 11.7% changed adherence category. At long-term assessment, device-recorded CPAP adherence, device-derived respiratory control (predefined residual apnea-hypopnea index [AHI] < 5 events/hour criterion), absence of residual sleepiness, and perceived treatment benefit were observed in 79.8%, 47.8%, 87.8%, and 89.3% of patients with available data, respectively. Among 71 patients with complete multidomain data, 45.1% simultaneously met all four predefined treatment-domain criteria, increasing to 63.4% using residual AHI < 10 events/hour. **Conclusions:** Care-pathway attendance and treatment outcomes represent distinct dimensions of long-term CPAP management. Similar aggregate adherence according to available device-recorded nights across assessments may conceal individual differences in adherence classification between observation intervals, while treatment outcomes are only partially concordant. Multidimensional reassessment may therefore identify clinically relevant discordance beyond adherence alone.

## 1. Introduction

Obstructive sleep apnea (OSA) is a highly prevalent chronic disorder affecting an estimated one billion adults worldwide [1,2]. Continuous positive airway pressure (CPAP) remains a standard treatment, particularly in symptomatic patients and those with clinically relevant disease [1,3]. By preventing upper-airway collapse during sleep, CPAP effectively reduces obstructive respiratory events and improves daytime sleepiness and sleep-related quality of life [3]. However, the benefits observed during CPAP use do not by themselves capture the complexity of long-term OSA management. In routine clinical practice, treatment is embedded within a longitudinal care process that extends from prescription and treatment initiation to continued engagement with follow-up, sustained device use, and reassessment of treatment outcomes over time [4]. Accordingly, evaluating long-term CPAP care requires consideration not only of treatment efficacy, but also of how patients progress through and remain engaged with the care pathway.

Long-term persistence with CPAP remains a major challenge. Large real-world studies have documented substantial treatment termination over time, with termination approaching half of treated patients by three years in a nationwide French cohort [5]. Longitudinal studies demonstrate heterogeneity in CPAP use, with some patients maintaining adherence over time, others remaining persistently non-adherent, and others changing adherence patterns during follow-up [6]. Importantly, treatment adherence and retention within a clinical care pathway represent related but distinct dimensions of long-term management. Patients may continue CPAP treatment without remaining under observation at the original center, whereas attendance at follow-up does not necessarily imply adequate device use [7]. Distinguishing care-pathway retention from treatment adherence may therefore provide a more informative description of real-world long-term CPAP management.

Treatment adherence alone, however, does not fully characterize the clinical status of patients receiving CPAP. Long-term treatment outcomes encompass complementary dimensions, including device use, control of residual respiratory events, symptom resolution, and perceived treatment benefit. These dimensions may diverge. Residual daytime sleepiness can persist despite adequate CPAP use and satisfactory control of respiratory events [8,9,10], while residual respiratory control may not closely track patient-centered outcomes [11]. Thus, adherence, device-derived respiratory control, symptoms, and perceived benefit provide related but non-interchangeable information, supporting a multidimensional assessment of long-term CPAP treatment [4,12].

Although long-term CPAP adherence, treatment discontinuation, residual symptoms, and patient-centered outcomes have each been investigated [5,8,13], these dimensions have generally been examined separately. Consequently, limited real-world evidence is available on their joint characterization within the same longitudinal care pathway. The present study therefore extends previous long-term CPAP research by evaluating care-pathway attendance separately from treatment status and, among patients with observed follow-up, characterizing the concordance and discordance across CPAP adherence, device-derived respiratory control, residual sleepiness, and perceived treatment benefit. On this basis, we hypothesized that long-term CPAP care involves two distinct but related dimensions: retention within the observed care pathway and treatment outcomes among patients remaining in follow-up. We further hypothesized that technical, device-derived respiratory-control, symptomatic, and patient-perceived treatment outcomes provide complementary, rather than interchangeable, information on long-term treatment status. Accordingly, the primary objective of this study was to characterize long-term follow-up attendance as an operational measure of retention in the observed CPAP care pathway. Secondary objectives were to describe longitudinal treatment outcomes among patients with paired follow-up data. We also characterized technical, device-derived respiratory-control, symptomatic, and patient-perceived treatment domains at long-term follow-up, including the number and combination of predefined criteria simultaneously met. Exploratory objectives were to examine factors associated with long-term follow-up attendance and pairwise associations among treatment-outcome domains.

## 2. Methods

### 2.1. Study Design and Population

This was a single-center observational study combining prospective data collection with retrospective extraction of previously recorded clinical data, conducted at the dedicated outpatient sleep clinic of the Respiratory Medicine Unit, Azienda Ospedaliera Universitaria Integrata of Verona, Italy. Consecutive adult patients (≥18 years) with OSA who were prescribed CPAP therapy between November 2019 and June 2026 were considered for inclusion. Patients with sleep-disordered breathing conditions other than OSA, technically inadequate diagnostic studies preventing reliable characterization of OSA, or insufficient clinical information for inclusion in the CPAP care pathway were excluded.

Patients entered the study cohort at the time of CPAP prescription. Because prescriptions occurred at different calendar dates, individual potential follow-up duration varied across the cohort. An administrative database closure date of 30 June 2026 was therefore used to determine potential follow-up duration and membership in the primary follow-up risk sets.

The study protocol was approved by the South-West Veneto Area Ethics Committee on 6 June 2025 (approval code 623CET). Retrospective screening and extraction of clinical data recorded before study approval were initiated only after Ethics Committee approval. Prospective data collection was conducted thereafter in accordance with the approved protocol. Written informed consent for participation in the study was obtained from all included patients. The study was conducted in accordance with the Declaration of Helsinki and Good Clinical Practice guidelines.

### 2.2. Clinical Variables and Comorbidities

Demographic characteristics, anthropometric measurements, relevant comorbidities, sleep-related clinical variables, and prescribed CPAP were retrospectively retrieved from the available medical records. Age was calculated from date of birth and the date of CPAP prescription, which represented the index date for entry into the observed CPAP care pathway. Body mass index (BMI) was calculated as weight in kilograms divided by height in meters squared, and obesity was defined as BMI ≥ 30 kg/m^2^.

Cardiovascular disease was defined as a documented history of cardiac disease, including ischemic heart disease, cardiomyopathic or dilated heart disease, and clinically documented cardiac arrhythmias. Other comorbidities, including diabetes mellitus, dyslipidemia, asthma, chronic obstructive pulmonary disease, renal disease, and history of malignancy, were identified from the clinical record.

### 2.3. Respiratory Polygraphy and Baseline OSA Assessment

Diagnostic respiratory polygraphy was performed using a portable Nox T3s^TM^ device (Nox Medical, Reykjavík, Iceland). Recordings included peripheral oxygen saturation (SpO_2_), thoracic and abdominal respiratory movements, derived respiratory signals, snoring, and body position. A minimum recording duration of 4 h with adequate signal quality was required for a technically valid study. Recordings were analyzed using Noxturnal^TM^ software, version 6.1 (Nox Medical, Reykjavík, Iceland). Automated respiratory-event scoring was reviewed and, when necessary, corrected by an experienced pulmonologist specialized in sleep medicine.

Respiratory events were scored according to American Academy of Sleep Medicine criteria [14]. Apnea was defined as a ≥90% reduction in airflow lasting ≥10 s and hypopnea as a ≥30% reduction in airflow lasting ≥10 s associated with a ≥3% oxygen desaturation. The apnea-hypopnea index (AHI) was calculated as the total number of apneas and hypopneas per hour of analyzed recording time. The oxygen desaturation index (ODI) was defined as the number of ≥3% oxygen desaturations per hour of analyzed recording time, and T_90_ as the percentage of analyzed recording time spent with SpO_2_ < 90%.

Daytime sleepiness at baseline was assessed using the validated Italian version of the Epworth Sleepiness Scale (ESS), an eight-item self-administered questionnaire yielding a total score from 0 to 24 [15]. An ESS score > 10 was considered indicative of excessive daytime sleepiness.

### 2.4. CPAP Treatment, Follow-Up Risk Sets, and Attendance

All patients entering the care pathway were prescribed fixed-pressure CPAP therapy. CPAP was prescribed in accordance with current clinical guidelines and routine clinical practice [3], taking into account OSA severity, symptoms, relevant comorbidities, and the overall clinical assessment of the treating sleep physician. Patients underwent a first follow-up assessment (T1) and a subsequent long-term follow-up assessment (T2), clinically scheduled approximately three years after CPAP prescription. Because follow-up timing reflected routine clinical practice, the actual interval from CPAP prescription to each assessment was recorded for each patient. No predefined calendar window around the nominal three-year time point was required for an observed assessment to be classified as T2; T2 was identified as the clinically recorded long-term follow-up assessment in the care pathway.

Primary follow-up risk sets were defined at the administrative database closure date (30 June 2026) according to potential follow-up duration from CPAP prescription and independently of observed attendance. The primary T1 risk set included patients with at least 12 months of potential follow-up. The primary T2 risk set included patients with at least 30 months of potential follow-up. This pragmatic operational threshold was selected to provide sufficient opportunity for the clinically scheduled approximately three-year assessment, rather than to define a fixed assessment window. Because the choice of threshold was operational, alternative minimum potential follow-up thresholds of 24 and 36 months were examined in sensitivity analyses (Supplementary Figure S1). Patients with shorter potential follow-up were considered not yet to have had sufficient opportunity to contribute to the corresponding primary attendance analysis and were therefore excluded from its denominator rather than classified as non-attenders, irrespective of whether an earlier assessment had already occurred. Such observed assessments could nevertheless contribute to treatment-outcome analyses. Accordingly, treatment-outcome analyses at T2 included all patients with an observed T2 assessment, irrespective of primary T2 risk-set membership. To assess whether inclusion of T2 assessments occurring before the ≥30-month potential follow-up threshold materially influenced the findings, a sensitivity analysis repeated the long-term treatment-outcome analyses after restriction to T2 attendees belonging to the primary ≥30-month risk set.

Within each primary risk set, attendance at the corresponding assessment was used to characterize retention in the observed CPAP care pathway. Absence of the corresponding assessment was classified as non-attendance and, for the long-term analysis, as non-retention in the observed care pathway. Non-retention was not interpreted as CPAP discontinuation or non-adherence, because CPAP use and follow-up outside the study center could not be fully ascertained.

### 2.5. Treatment-Outcome Definitions

CPAP adherence was objectively assessed from device-recorded usage data downloaded at each follow-up assessment. At each visit, all usage data stored by the CPAP device since the preceding clinical assessment were reviewed; for T1, this corresponded to the interval from CPAP treatment initiation to the first follow-up, whereas for T2 it corresponded to the interval since the preceding follow-up assessment. Thus, adherence at T1 and T2 referred to distinct, visit-specific observation intervals of potentially different duration and should not be interpreted as measurements over standardized or identical exposure windows. Patients were classified as adherent when CPAP was used for ≥4 h/night on ≥70% of recorded nights during the corresponding observation interval [6,16]. The available device reports categorized recorded nights according to CPAP use ≥4 or <4 h. Complete coverage of every calendar night within each observation interval, including nights with no device use or no stored data, could not be retrospectively verified. Accordingly, adherence was calculated using the device-recorded nights available in the clinical report rather than assuming complete calendar-night coverage. Patients with available device data who did not meet this criterion were classified as non-adherent, whereas patients without available device-derived adherence information were considered to have missing adherence data.

Residual AHI at T1 and T2 was obtained from device-derived CPAP data downloaded during the corresponding follow-up assessment and represented the respiratory-event index reported by the device for the corresponding observation period, rather than a single-night measurement. Thus, residual AHI reflected respiratory events detected by the CPAP device during treatment and was distinct from the baseline diagnostic AHI derived from respiratory polygraphy. Patients were treated with fixed-pressure CPAP, but device models were not uniform across the cohort. No change in treatment modality or device replacement was recorded during follow-up. Changes in prescribed CPAP pressure during follow-up were not systematically captured in the study dataset and could therefore not be evaluated. Device-derived leak data and separate indices for residual obstructive and central respiratory events were not systematically collected and were therefore unavailable for analysis.

Residual sleepiness was assessed during each follow-up visit by the physician conducting the clinical assessment and was recorded as a binary outcome (present/absent). Patients were directly asked whether daytime sleepiness persisted during CPAP treatment, and this information was integrated with the overall clinical assessment. When available, the ESS contributed to, but did not solely determine, the classification. The same routine clinical approach was used at both T1 and T2. The assessment was performed as part of routine clinical practice and did not follow a predefined standardized algorithm. No ambiguous or discordant classifications were documented in the medical records. Residual sleepiness and ESS score were therefore treated as related but distinct variables.

Perceived treatment benefit was assessed during the clinical encounter using a direct yes/no question asking patients whether they perceived benefit from CPAP treatment and was recorded as a binary variable (present/absent). The question addressed overall perceived treatment benefit rather than a specific domain such as sleep quality, daytime functioning, symptoms, or health-related quality of life, and was not based on a validated patient-reported outcome instrument. The same assessment approach was used at both follow-up visits. When perceived treatment benefit was not documented in the clinical record, it was treated as missing rather than as absence of perceived benefit.

Four treatment-outcome domains were evaluated at long-term follow-up: technical, defined as device-derived CPAP adherence; device-derived respiratory control, defined using a predefined residual AHI < 5 events/hour; symptomatic, defined as absence of clinically assessed residual sleepiness; and patient-perceived, defined as the patient’s subjective report of benefit from CPAP treatment. The residual AHI < 5 events/hour threshold was selected as a stringent operational criterion reflecting normalization of the device-reported respiratory-event index and was not intended to represent a universally accepted definition of CPAP treatment success. Because less stringent residual AHI thresholds may also be clinically acceptable depending on the individual context, a sensitivity analysis used residual AHI < 10 events/hour to assess the dependence of the multidomain findings on the operational definition of respiratory control. The domains were analyzed individually and descriptively according to the number and combination of predefined criteria simultaneously met and were not combined into a validated composite treatment endpoint.

### 2.6. Statistical Analysis

For the purposes of analysis and reporting, study outcomes were organized according to a conceptual hierarchy. Long-term follow-up attendance within the primary T2 risk set was treated as the primary descriptive endpoint, as an operational measure of retention in the observed CPAP care pathway. Secondary analyses characterized treatment outcomes among patients with available long-term data and within-patient paired comparisons among patients with measurements available at both assessments. Regression analyses of factors associated with long-term follow-up attendance and pairwise associations among treatment-outcome domains were considered exploratory.

Continuous variables were summarized according to their observed distribution as mean ± standard deviation (SD) when approximately normally distributed and median [interquartile range (IQR)] otherwise. Categorical variables were reported as counts and percentages, with denominators explicitly reported when data were missing.

Baseline characteristics were compared between patients who attended and did not attend T2 within the primary T2 risk set, defined as patients with at least 30 months of potential follow-up at database closure. Student’s *t*-test was used for approximately normally distributed continuous variables, the Mann–Whitney U test for non-normally distributed continuous variables, and the chi-square test or Fisher’s exact test for categorical variables, as appropriate. These comparisons were descriptive and were not used for data-driven variable selection.

Patient progression through the observed CPAP care pathway was described using primary risk sets defined exclusively by potential follow-up duration. The primary T1 and T2 attendance denominators comprised patients with at least 12 and 30 months of potential follow-up, respectively, at administrative database closure. Patients below the corresponding threshold were reported separately and excluded from the primary attendance denominator irrespective of observed attendance. Long-term follow-up attendance was additionally evaluated using alternative minimum potential follow-up thresholds of 24 and 36 months as sensitivity analyses. A 95% confidence interval (CI) for the primary T2 attendance proportion was calculated using the Wilson method. Adherence prevalence at long-term follow-up was calculated among patients with available device-derived adherence data in the observed T2 assessment cohort; patients without adherence data were not classified as non-adherent. Time from CPAP prescription to follow-up assessments was summarized as median [IQR] among patients who actually underwent the corresponding assessment.

Longitudinal analyses included only patients with the corresponding outcome available at both T1 and T2; accordingly, descriptive values at T1 and T2 were calculated within the same paired cohort for each outcome. Continuous paired outcomes were compared using the Wilcoxon signed-rank test and binary outcomes using McNemar’s test. Effect estimates were additionally reported as median within-patient differences for continuous outcomes and absolute paired differences in proportions for binary outcomes, with 95% CIs obtained using 50,000 paired bootstrap resamples. Bootstrap resampling was performed at the patient level, preserving paired T1–T2 observations within each resampled subject. Paired samples comprised 94 patients for CPAP adherence, 85 for residual AHI and residual sleepiness, 68 for ESS score, and 67 for perceived treatment benefit. Individual transitions between adherence categories across the two observation intervals were also described.

Individual treatment-outcome proportions at long-term follow-up were calculated using patients with available data for each domain as the denominator. Analyses of the number and combination of treatment-domain criteria simultaneously met were restricted to the 71 patients with complete data for all four domains. These analyses were descriptive and were not intended to define or validate a composite treatment endpoint. The multidomain analysis was repeated using residual AHI < 10 events/hour as an alternative definition of device-derived respiratory control; no formal statistical comparison between AHI thresholds was performed. As an additional sensitivity analysis, all long-term treatment-outcome estimates were recalculated after restricting the observed T2 cohort to patients belonging to the primary ≥30-month T2 risk set.

Factors associated with long-term follow-up attendance were explored using logistic regression within the primary T2 risk set, defined as patients with at least 30 months of potential follow-up at database closure. Results were reported as odds ratios (ORs) with 95% confidence intervals (CIs). Univariate models were fitted separately for age, sex, BMI, baseline AHI, baseline T90, baseline ESS score, and cardiovascular disease. Continuous predictors were scaled per 10-year increase in age, 5 kg/m^2^ increase in BMI, 10-events/hour increase in AHI, 10-percentage-point increase in T_90_, and 5-point increase in ESS score. To limit model complexity given the number of non-attendance events and incomplete availability of several baseline covariates, the adjusted analysis was deliberately parsimonious and included age and sex only. The age- and sex-adjusted model included the entire primary T2 risk set (n = 114; 80 attended T2 and 34 did not attend T2), as age and sex were available for all patients. Given the limited number of non-attendance events and the exploratory nature of these analyses, regression estimates were interpreted as associations with attendance at the study center rather than as predictive models of CPAP persistence or adherence.

The sensitivity of long-term attendance estimates and the age association to the definition of the T2 risk set was examined using alternative minimum potential follow-up thresholds of 24 and 36 months. In the primary ≥ 30-month risk set, an additional sensitivity model including age, sex, and potential follow-up duration was fitted to assess whether variation in follow-up opportunity influenced the age estimate. Attendance was also described according to calendar year of CPAP prescription. Because very few patients in the primary T2 risk set had been prescribed CPAP in 2019 or 2020, annual attendance estimates were considered descriptive and no formal comparison across individual calendar years was performed. A further sensitivity model including age, sex, and calendar year of CPAP prescription was fitted to explore potential calendar-period effects.

Treatment characteristics measured at T1—CPAP adherence, residual AHI, residual sleepiness, and perceived treatment benefit—were explored among patients who attended T1 and belonged to the primary T2 risk set (n = 102; 80 attended T2 and 22 did not attend T2). Each treatment characteristic was evaluated in a separate univariate logistic regression model and subsequently in a separate model adjusted for age. Models were based on available cases for the corresponding treatment characteristic and age.

Pairwise associations among the four binary treatment-outcome domains were evaluated in the complete-case–cohort (n = 71) using the phi coefficient (ϕ), with Fisher’s exact test used because of small expected cell counts. These analyses were exploratory, and *p*-values were not adjusted for multiple comparisons. Phi coefficients were interpreted descriptively and not as evidence of statistical independence between domains.

Cross-sectional analyses used available cases and longitudinal analyses used complete pairs. Data availability is detailed in Supplementary Table S1. All tests were two-sided. Statistical analyses were performed using IBM SPSS Statistics version 30 (IBM Corp., Armonk, NY, USA).

## 3. Results

### 3.1. Study Population and Long-Term CPAP Care Pathway

**Figure 1:**
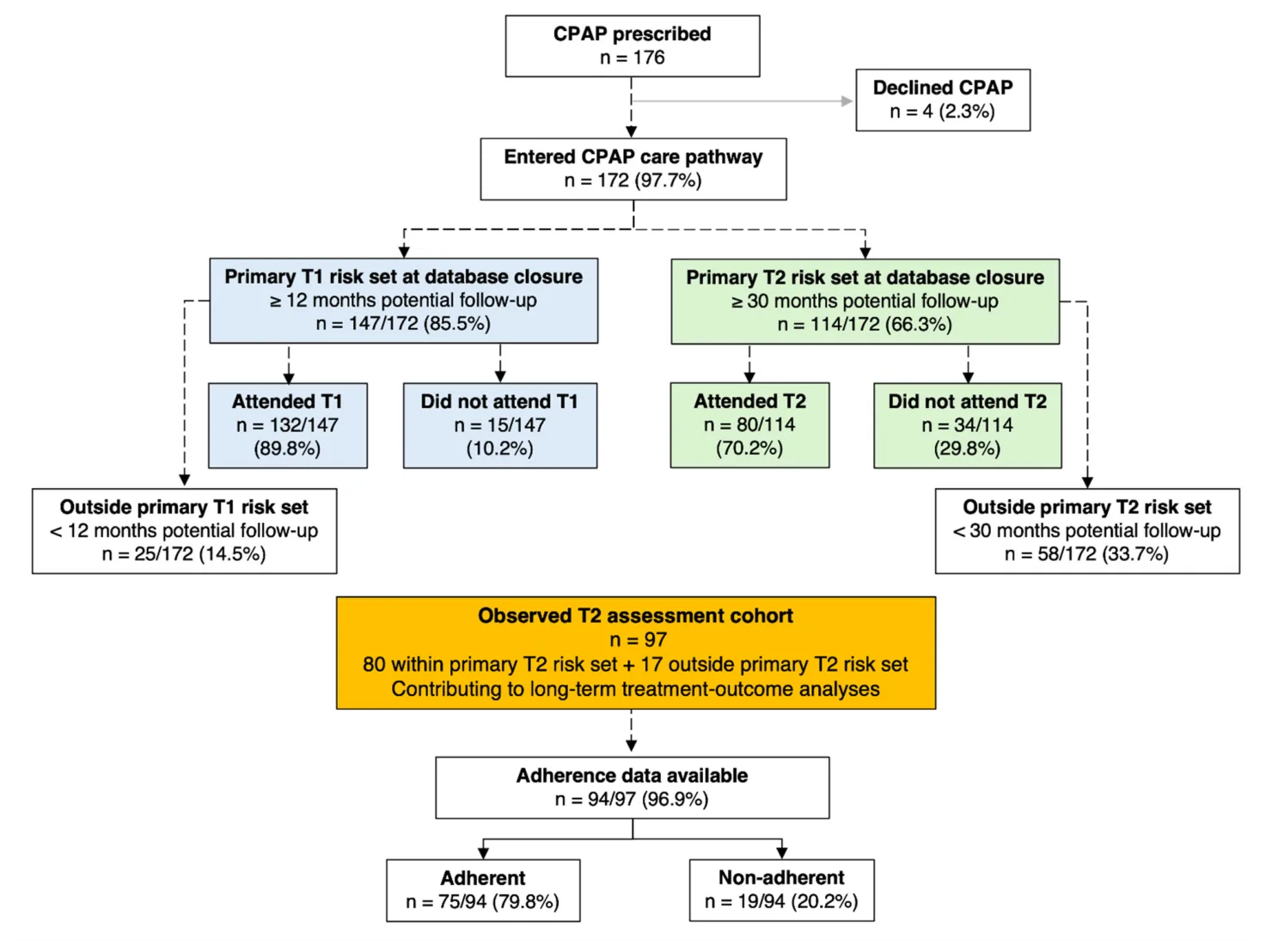
Patient flow, follow-up risk sets, and attendance in the observed CPAP care pathway. The flow diagram illustrates patient progression from CPAP prescription to follow-up attendance and long-term treatment assessment. Of 176 patients prescribed CPAP, 4 declined treatment and 172 entered the observed CPAP care pathway. Primary follow-up risk sets were defined independently of observed attendance according to potential follow-up duration from CPAP prescription to the administrative database closure date (30 June 2026). The primary T1 risk set comprised 147 patients with at least 12 months of potential follow-up, of whom 132 (89.8%) attended T1, and 15 (10.2%) did not. The primary T2 risk set comprised 114 patients with at least 30 months of potential follow-up, of whom 80 (70.2%) attended T2, and 34 (29.8%) did not. Patients below the corresponding potential follow-up threshold were excluded from the primary attendance denominators irrespective of whether an early assessment had occurred. Overall, 97 patients underwent T2 and constituted the observed long-term assessment cohort contributing to treatment-outcome analyses. This cohort comprised 80 patients who attended T2 within the primary T2 risk set and 17 patients who underwent T2 despite having <30 months of potential follow-up at database closure and were therefore outside the primary T2 risk set. Among the 97 patients with an observed T2 assessment, device-derived adherence data were available for 94, of whom 75 (79.8%) were adherent, and 19 (20.2%) were non-adherent. Patients without available adherence data were not classified as non-adherent. Non-attendance at the study center was not interpreted as CPAP discontinuation or absence of OSA care elsewhere. Abbreviations: CPAP, continuous positive airway pressure; T1, first follow-up assessment; T2, long-term follow-up assessment.

A total of 176 patients were prescribed CPAP. Four (2.3%) declined treatment, leaving 172 patients who entered the observed CPAP care pathway. For the first follow-up assessment, 147/172 patients (85.5%) had at least 12 months of potential follow-up at database closure and constituted the primary T1 risk set. Of these, 132 (89.8%) attended T1 and 15 (10.2%) did not. Twenty-five patients had <12 months of potential follow-up and were therefore outside the primary T1 risk set. For the long-term assessment, 114/172 patients (66.3%) had at least 30 months of potential follow-up and constituted the primary T2 risk set. Of these, 80 (70.2%; 95% CI 61.2–77.8%) attended T2 and 34 (29.8%) did not. Fifty-eight patients had <30 months of potential follow-up and were outside the primary T2 risk set (Figure 1).

Overall, 136 patients underwent T1 and 97 underwent T2, including assessments occurring outside the corresponding primary risk sets. The 97 patients with an observed T2 assessment constituted the long-term assessment cohort contributing to treatment-outcome analyses. Of these 97 patients, 80 belonged to the primary T2 risk set, and 17 underwent T2 despite having <30 months of potential follow-up at database closure. The 17 assessments occurring outside the primary T2 risk set were included in observed treatment-outcome analyses because a T2 assessment had actually been performed, whereas the primary T2 risk set was used specifically for the attendance analysis. The first follow-up occurred at a median of 357 days [IQR 203–395] after CPAP prescription, whereas the long-term assessment occurred at a median of 979 days [IQR 763–1143]. Among the 97 patients with an observed T2 assessment, adherence data were available for 94, of whom 75 (79.8%) were adherent.

Baseline characteristics according to long-term follow-up attendance are reported in Table 1. Patients who attended T2 were older than those who did not (69.1 [59.3–75.0] vs. 59.2 [50.1–72.7] years; *p* = 0.018). Apart from age, estimates for other baseline characteristics were imprecise and did not provide clear evidence of between-group differences.

### 3.2. Longitudinal Treatment Outcomes

**Figure 2:**
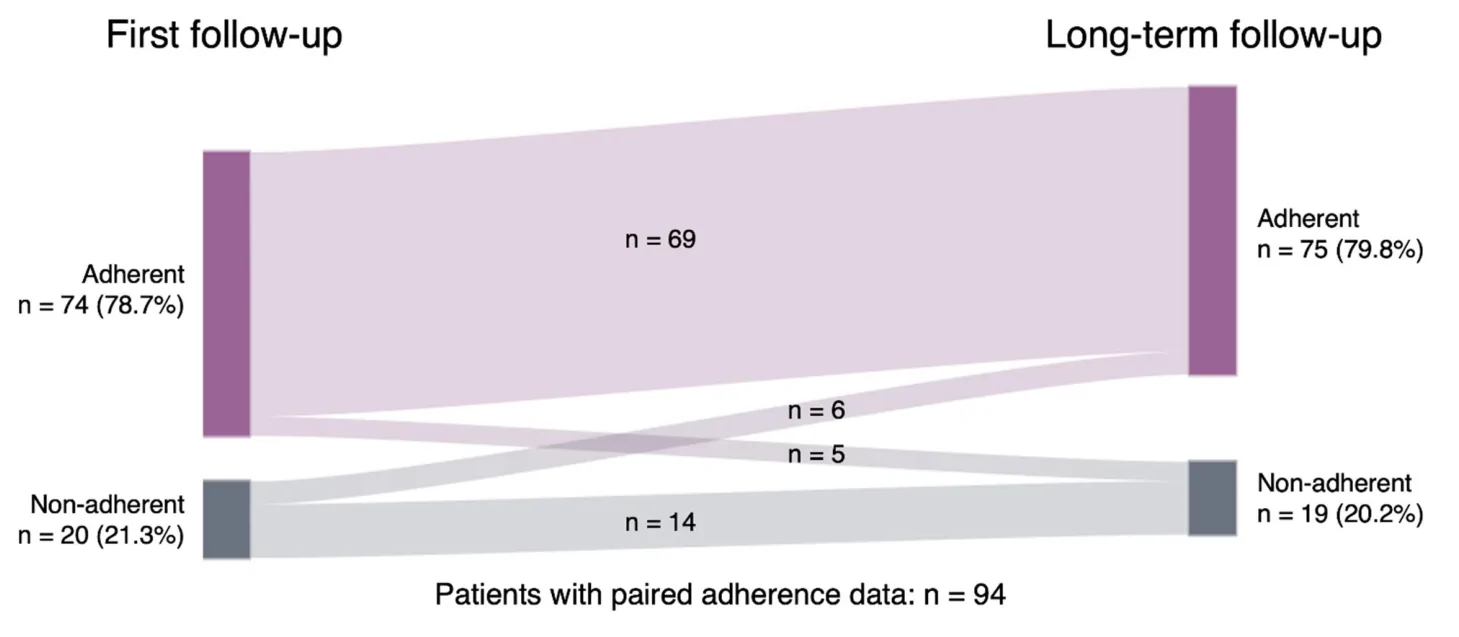
Sankey diagram of individual CPAP adherence categories at the first and long-term follow-up assessments among patients with paired adherence data (n = 94). Adherence at each assessment was defined as CPAP use for ≥4 h/night on ≥70% of recorded nights during the corresponding visit-specific observation interval. Overall adherence prevalence was 78.7% at T1 and 79.8% at T2. Eighty-three patients (88.3%) remained in the same adherence category across the two observation intervals, whereas 11 (11.7%) changed category: 5 were classified as adherent at T1 and non-adherent at T2, and 6 as non-adherent at T1 and adherent at T2. T1 and T2 adherence were derived from distinct observation intervals and should not be interpreted as measurements over identical exposure windows.

Among patients with paired outcome data, measured treatment outcomes showed little evidence of systematic differences between assessments (Table 2). Among the 94 patients with paired adherence data, the median interval between T1 and T2 was 677 days [IQR 404–775], and T2 occurred at a median of 977 days after CPAP prescription [IQR 758–1151]. Adherence according to available device-recorded nights was similar across the two visit-specific observation intervals: 78.7% at T1 and 79.8% at T2 (paired difference +1.1 percentage points, 95% CI −5.3 to +8.5; McNemar *p* = 1.000). Despite this similar aggregate prevalence, 83/94 patients (88.3%) remained in the same adherence category across the two observation intervals, whereas 11 (11.7%) changed category, including 5 classified as adherent at T1 but non-adherent at T2 and 6 classified as non-adherent at T1 but adherent at T2 (Figure 2).

The median within-patient change in residual AHI was +0.2 events/hour (95% CI −0.2 to +0.8; paired n = 85; *p* = 0.222), while the median within-patient change in ESS score was 0 points (95% CI −1 to 0; paired n = 68; *p* = 0.368). Paired differences were also small for residual sleepiness (0.0 percentage points, 95% CI −8.2 to +8.2; paired n = 85; *p* = 1.000) and perceived treatment benefit (−1.5 percentage points, 95% CI −9.0 to +6.0; paired n = 67; *p* = 1.000).

### 3.3. Multidomain Treatment Outcomes at Long-Term Follow-Up

At long-term follow-up, 75/94 patients (79.8%) met the technical outcome of CPAP adherence, 43/90 (47.8%) met the predefined device-derived respiratory-control criterion of residual AHI < 5 events/hour, 79/90 (87.8%) met the symptomatic outcome of absence of clinically assessed residual sleepiness, and 67/75 (89.3%) reported perceived treatment benefit (Table 3).

**Figure 3:**
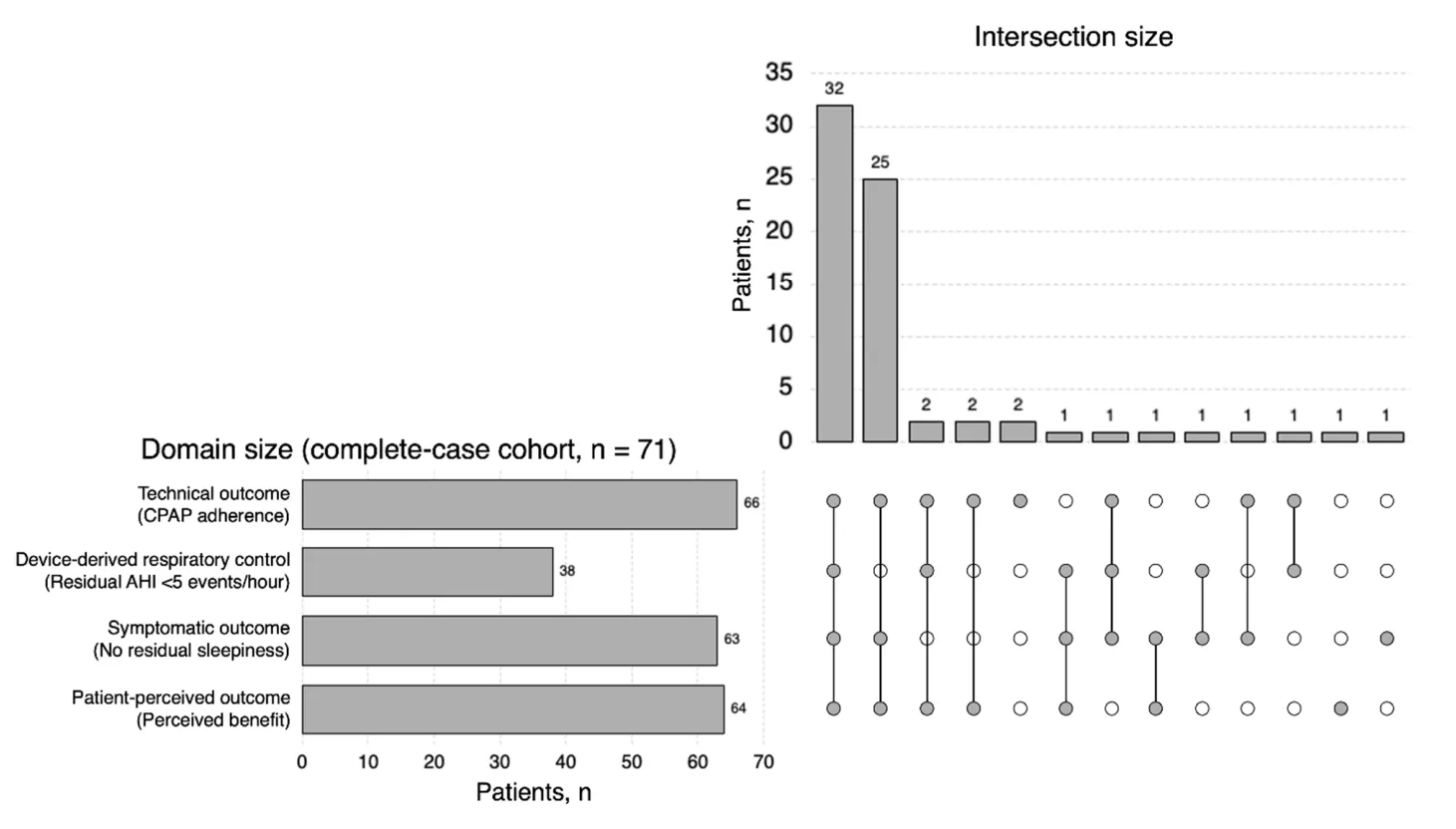
Overlap of CPAP treatment-outcome domains at the long-term follow-up (complete-case–cohort, n = 71). The UpSet plot illustrates the overlap among the four treatment-outcome domains in the 71 patients with complete data for all domains. The analysis was restricted to the 71 patients with complete data for all four treatment domains and should therefore be interpreted as a complete-case description rather than as representing the entire observed T2 cohort. Characteristics of patients with complete versus incomplete multidomain data are reported in Supplementary Table S2. Technical outcome was defined as device-derived CPAP adherence (≥4 h/night on ≥70% of nights); device-derived respiratory control as a residual apnea-hypopnea index (AHI) < 5 events/hour; symptomatic outcome was defined as the absence of clinically assessed residual sleepiness; and patient-perceived outcome was based on the patient’s subjective report of benefit from CPAP treatment during the clinical follow-up assessment. Horizontal bars indicate the total number of patients meeting each individual domain, while vertical bars indicate the number of patients meeting each specific combination of domains. Filled circles identify domain criteria met within each combination, whereas open circles indicate criteria not met. Thirty-two patients (45.1%) simultaneously met all four predefined treatment-domain criteria, whereas 25 (35.2%) met technical, symptomatic, and patient-perceived criteria without meeting the device-derived respiratory control criterion.

Among the 71 patients with complete data for all four domains, 67 (94.4%) met criteria for at least two domains, 61 (85.9%) for at least three domains, and 32 (45.1%) simultaneously met all four predefined treatment-domain criteria. The most frequent discordant pattern comprised 25 patients (35.2%) who met the technical, symptomatic, and patient-perceived outcomes but not the device-derived respiratory-control criterion (Figure 3).

The multidomain profile was sensitive to the operational definition of device-derived respiratory control. Using residual AHI < 10 rather than <5 events/hour increased the proportion meeting the device-derived respiratory-control criterion from 47.8% to 77.8% and the proportion simultaneously meeting all four predefined treatment-domain criteria from 45.1% to 63.4%.

Results were materially unchanged when long-term treatment-outcome analyses were restricted to the 80 T2 attendees belonging to the primary ≥ 30-month risk set. In this restricted cohort, CPAP adherence was observed in 62/77 patients (80.5%), residual AHI < 5 events/hour in 34/75 (45.3%), absence of residual sleepiness in 63/74 (85.1%), and perceived treatment benefit in 53/61 (86.9%). Among the 58 patients with complete data for all four domains, 24 (41.4%) simultaneously met all four predefined domain criteria using residual AHI < 5 events/hour, increasing to 36/58 (62.1%) when residual AHI < 10 events/hour was used. Detailed results are reported in Supplementary Table S3.

Among the 97 patients with an observed T2 assessment, 71 had complete data for all four treatment-outcome domains and therefore contributed to the complete-case multidomain analysis, whereas 26 had incomplete multidomain data. Perceived treatment benefit was the treatment domain with the greatest amount of missing data at T2, being documented in 75/97 patients (77.3%); absence of documentation was treated as missing rather than as absence of perceived benefit (Supplementary Table S1). Patients included in the complete-case–cohort were more frequently adherent at T1 than those with incomplete multidomain data (88.7% vs. 50.0%) and showed some descriptive differences in baseline characteristics, particularly BMI (Supplementary Table S2). These findings indicate potential selection into the complete-case multidomain cohort; accordingly, the proportion simultaneously meeting all four domain criteria should be interpreted as applying to the 71 complete cases rather than to the entire observed T2 cohort.

### 3.4. Factors Associated with Long-Term Follow-Up Attendance

Within the primary T2 risk set, older age was associated with greater long-term follow-up attendance in univariate logistic regression (OR 1.52 per 10-year increase; 95% CI 1.06–2.18; *p* = 0.022), whereas estimates for the remaining baseline factors did not provide clear evidence of association (Table 4). In the parsimonious age- and sex-adjusted model (n = 114), the estimate for age remained similar (adjusted OR 1.58 per 10-year increase; 95% CI 1.10–2.28; *p* = 0.014), while the estimate for male sex was imprecise (adjusted OR 2.35; 95% CI 0.81–6.82; *p* = 0.115).

Long-term follow-up attendance was similar when alternative minimum potential follow-up thresholds of 24 months (89/130, 68.5%) and 36 months (67/92, 72.8%) were applied, compared with 80/114 (70.2%) using the primary 30-month threshold (Supplementary Table S4). The age estimate was essentially unchanged after additional adjustment for potential follow-up duration (adjusted OR 1.58 per 10-year increase; 95% CI 1.10–2.29; *p* = 0.014), whereas potential follow-up duration itself showed no clear association with T2 attendance (adjusted OR 1.00 per year; 95% CI 0.63–1.57; *p* = 0.995). Age estimates were directionally consistent across alternative 24- and 36-month risk-set definitions, although precision was reduced at the 36-month threshold (Supplementary Table S4).

No clear monotonic pattern in long-term attendance was observed across calendar years of CPAP prescription. In a sensitivity model additionally accounting for calendar year, the age estimate remained essentially unchanged (adjusted OR 1.59 per 10-year increase; 95% CI 1.10–2.31), whereas calendar year showed no clear association with T2 attendance (Supplementary Table S4).

Among patients who attended T1 and belonged to the primary T2 risk set, no clear evidence was observed that T1 adherence, residual AHI, residual sleepiness, or perceived treatment benefit was associated with subsequent T2 attendance after adjustment for age; estimates were imprecise, particularly for binary clinical outcomes (Table 4).

### 3.5. Relationships Among Treatment-Outcome Domains

Pairwise associations among treatment-outcome domains were generally weak (Table 5). Device-derived respiratory control showed weak associations with technical (ϕ = 0.075), symptomatic (ϕ = 0.114), and patient-perceived (ϕ = 0.071) outcomes. The largest observed pairwise association was between the symptomatic and patient-perceived outcomes (ϕ = 0.330; nominal *p* = 0.027), followed by the technical and patient-perceived outcomes (ϕ = 0.278; nominal *p* = 0.073). These analyses were exploratory, and nominal *p*-values were not adjusted for multiple comparisons.

## 4. Discussion

In this real-world cohort of patients with OSA prescribed CPAP, long-term management was characterized by two distinct but complementary dimensions: continued engagement with the observed care pathway and treatment status among patients who remained under assessment. Approximately 70% of patients with sufficient follow-up opportunity attended the long-term assessment. Treatment-outcome estimates were also materially unchanged when analyses were restricted to T2 attendees within the primary ≥30-month risk set. Among patients with observed long-term outcome data, CPAP adherence according to available device-recorded nights was similar across the two visit-specific observation intervals. However, individual adherence classification differed between intervals in a subset of patients. Treatment outcomes were only partially concordant across treatment-domain criteria: the technical, symptomatic, and patient-perceived criteria were frequently met, whereas the stringent device-derived respiratory-control criterion of residual AHI < 5 events/hour was less frequently met. Taken together, these findings support a view of long-term CPAP management in which care-pathway engagement, treatment adherence, device-derived respiratory control, symptoms, and perceived benefit provide complementary rather than interchangeable information [4,12]. By examining care-pathway attendance separately from treatment status and then evaluating multiple treatment domains within the same observed cohort, this study provides an integrated perspective that complements previous long-term CPAP studies focused predominantly on individual endpoints.

Long-term follow-up attendance represents a distinct aspect of CPAP management that should not be conflated with treatment persistence or device adherence. Direct comparison of our approximately 70% long-term attendance with previously reported CPAP continuation or termination rates is difficult because previous studies have generally used treatment-based rather than care-pathway-based endpoints. For example, CPAP termination approached half of treated patients at three years in a large French nationwide analysis, but was defined according to cessation of CPAP reimbursement [5]. Patients may also follow heterogeneous trajectories after CPAP initiation, including continued treatment at the original center, transfer to another facility, treatment discontinuation, or other circumstances leading to cessation of follow-up at the original center [7]. Thus, device persistence, treatment persistence, and retention within a specific care pathway represent related but non-equivalent constructs. Older age was associated with greater long-term follow-up attendance in our cohort. The direction of this association was consistent after accounting for sex, potential follow-up duration, and calendar year and across alternative risk-set definitions, although precision was reduced with the most restrictive 36-month threshold. Previous observational studies have also linked age and other demographic or clinical characteristics to CPAP persistence or adherence [5,13,17]. However, these associations have not been uniform across populations, and baseline characteristics alone appear to have limited predictive ability. Importantly, this exploratory association concerns attendance within the study center rather than CPAP adherence or treatment persistence. Given the limited number of non-attendance events, the age estimate should be interpreted cautiously and should not be regarded as evidence that age independently predicts CPAP persistence or should define follow-up intensity.

Among patients with paired adherence data, CPAP adherence according to available device-recorded nights was nearly identical across the two visit-specific observation intervals. Because T1 and T2 adherence were derived from distinct and potentially unequal observation periods, this finding should not be interpreted as demonstrating stability of cumulative adherence over time. Nevertheless, the similar aggregate prevalence is consistent with previous evidence that adherence patterns may become established relatively early after CPAP initiation and subsequently persist [6]. In a large telemonitored cohort, adherence patterns largely stabilized within the first three months, with a high probability of remaining in either the highly adherent or non-adherent state at two years [6]. Our individual-level analysis adds an important nuance: despite similar aggregate adherence prevalence, 11.7% of patients changed adherence category between the two observation intervals, with transitions occurring in both directions. These transitions should likewise be interpreted as differences in interval-specific adherence classification rather than necessarily as changes in cumulative long-term adherence status. Other cohorts have reported declining CPAP use or adherence over longer follow-up [17,18]. Together, these observations support repeated assessment of adherence during longitudinal follow-up rather than reliance on a single cross-sectional measurement.

A central finding of this study was the incomplete concordance among different dimensions of long-term CPAP treatment outcome. The technical, symptomatic, and patient-perceived criteria were met by approximately 80–90% of patients with available data, whereas fewer than half met the stringent device-derived respiratory-control criterion of residual AHI < 5 events/hour. Among patients with complete multidomain data, the most frequent discordant pattern consisted of meeting the technical, symptomatic, and patient-perceived criteria without meeting the device-derived respiratory-control criterion. This discordance was also reflected in the weak pairwise associations between device-derived respiratory control and the other treatment domains. The symptomatic and patient-perceived outcomes showed the largest observed pairwise association, although this finding was exploratory and based on an unadjusted nominal *p*-value. These findings are consistent with the broader recognition of OSA as a heterogeneous disorder whose clinical impact and response to treatment cannot be adequately represented by a single physiological or symptomatic metric [4,12,19]. Sex-related differences further contribute to this heterogeneity. Previous studies have documented differences between men and women in anatomical characteristics associated with upper-airway obstruction and in the clinical presentation and symptom reporting of OSA [20,21]. These differences may influence how treatment-related symptoms and patient-perceived outcomes are interpreted, although sex-specific treatment responses were not evaluated in the present study. More broadly, physiological and patient-centered outcomes do not necessarily improve in parallel. CPAP may produce greater reductions in AHI than alternative therapies without proportionally greater improvements in sleepiness or quality of life [22], while residual AHI showed no clear association with concurrent patient-reported outcomes after CPAP treatment [11]. Conversely, residual sleepiness may persist despite apparently adequate CPAP treatment and may reflect multiple contributing factors beyond adherence or residual respiratory events alone [8,9]. Together, these observations support assessing adherence, residual respiratory burden, symptoms, and perceived benefit as complementary rather than interchangeable dimensions of treatment response.

The degree of multidomain concordance was strongly influenced by the operational definition of device-derived respiratory control. When the residual AHI threshold was changed from <5 to <10 events/hour, the proportion simultaneously meeting all four predefined treatment-domain criteria increased from 45.1% to 63.4%. This threshold dependence is important when interpreting residual AHI as a marker of treatment effectiveness. Accordingly, the <5 events/hour threshold used in the primary analysis should be regarded as a stringent operational criterion rather than a universally accepted boundary between adequate and inadequate CPAP treatment. Interpretation of elevated device-reported residual AHI is further limited by heterogeneity in device models and their event-detection algorithms and by the absence of systematically available data on pressure changes, leak, and residual event type. Although no change in treatment modality or device replacement was recorded during follow-up, these limitations preclude detailed attribution of elevated residual AHI to pressure-related factors, leak, residual obstructive or central events, or device-related measurement differences. More broadly, residual respiratory events represent only one component of CPAP assessment, and AHI alone does not fully capture OSA burden or its impact on the individual [12]. Accordingly, our four-domain framework was intended to describe heterogeneity in treatment outcomes rather than to define a composite measure of CPAP success. Simultaneous meeting all four predefined treatment-domain criteria should not be interpreted as a validated global treatment endpoint, because the domains were not weighted according to clinical importance and the device-derived respiratory-control component itself depends on the threshold applied. Rather than defining treatment success by the number of predefined criteria met, the framework illustrates how treatment status may differ according to the outcome dimension and operational definition being considered.

These findings have practical implications for the long-term follow-up of patients receiving CPAP. A device-centered assessment based predominantly on adherence and residual AHI may fail to identify clinically relevant discordance between treatment use, device-derived respiratory control, residual symptoms, and perceived benefit. Long-term reassessment should therefore integrate device-derived information with symptoms and the patient’s experience of treatment [4,23]. This does not necessarily imply uniform in-person follow-up for all patients; contemporary models increasingly combine remote CPAP monitoring with targeted clinical reassessment according to individual needs and emerging treatment problems [23,24]. Persistent symptoms despite adequate adherence should prompt evaluation for residual OSA and other potentially modifiable contributors, including insomnia, comorbid conditions, medications, and other clinical factors [25]. Conversely, an elevated device-derived residual AHI in an otherwise adherent and clinically improved patient should prompt appropriate reassessment rather than being interpreted in isolation as global treatment failure. From this perspective, the clinical value of multidomain assessment may lie less in counting the number of predefined criteria met than in identifying discordant profiles that may prompt individualized reassessment and point to different clinical needs. Prospective studies are needed to determine whether follow-up strategies explicitly incorporating such multidimensional profiles improve clinical outcomes or healthcare efficiency.

This study has several strengths. First, the cohort was assembled from the time of CPAP prescription, allowing the care pathway to be examined separately from treatment outcomes among patients subsequently observed at follow-up. Second, follow-up risk sets were defined according to potential follow-up duration and independently of observed attendance, thereby separating administrative opportunity for follow-up from actual attendance; the long-term attendance estimate was also consistent across alternative risk-set definitions. Third, the use of outcome-specific paired analyses, device-derived treatment data, and a multidomain framework allowed within-patient longitudinal comparisons and individual transitions to be distinguished from cross-sectional treatment status.

Several limitations should nevertheless be considered. First, this was a single-center observational study, and part of the data collection relied on retrospective extraction of previously recorded clinical data. The single-center setting and the characteristics of the local CPAP care pathway may limit the generalizability of the observed attendance patterns and treatment-outcome profile to other healthcare settings, follow-up models, and patient populations. Furthermore, the marked predominance of men in the primary long-term follow-up risk set (83.3%) limits the generalizability of our findings to women, who may differ in OSA phenotype, symptom presentation, and treatment-related characteristics. The relatively small number of women would have resulted in imprecise sex-stratified estimates and therefore limited meaningful sex-specific analyses. The operational definition of long-term retention reflected attendance within the study center rather than persistence with CPAP treatment or continued OSA care elsewhere. The 30-month threshold used to define the primary T2 risk set was pragmatic rather than a fixed clinical time point; however, attendance estimates were similar using alternative thresholds of 24 and 36 months. Because scheduled appointment dates were not systematically available, we could not determine whether some patients classified as non-attenders after ≥30 months of potential follow-up were still awaiting their clinically scheduled T2 assessment at database closure. The primary 70.2% attendance estimate should therefore be interpreted in light of this potential misclassification; reassuringly, attendance was similar when the more restrictive ≥36-month threshold was applied (72.8%). Potential follow-up duration and calendar period may also have influenced attendance, although sensitivity analyses accounting for these factors did not materially alter the age association. Because study inclusion required written informed consent, some degree of consent-related selection cannot be excluded.

Second, treatment-outcome analyses were conditional on observed follow-up and data availability. Although 97 patients underwent T2, only 71 had complete data for all four treatment domains. Patients with complete multidomain data differed descriptively from those with incomplete data, most notably in T1 adherence, which was substantially more frequent among complete cases. Consequently, the proportion simultaneously meeting all four predefined treatment-domain criteria (45.1%) applies specifically to the 71 patients with complete data and may not represent the entire observed T2 cohort. Selection associated with follow-up attendance and outcome completeness may therefore have influenced the observed long-term treatment profile. The overall sample size, and particularly the complete-case multidomain cohort of 71 patients, also limited statistical precision and the ability to detect modest associations between treatment domains. Furthermore, regression analyses of factors associated with attendance were exploratory and based on a limited number of non-attendance events (n = 34), which restricted model complexity and resulted in imprecise estimates for several associations.

Third, several outcome measures reflected routine clinical practice rather than standardized research assessments. CPAP adherence was derived from device-recorded data collected over observation intervals determined by routine follow-up timing, and complete coverage of every calendar night within each interval could not be retrospectively verified. Consequently, adherence estimates reflect the nights represented in the available device reports and may not capture periods with no stored device data. Residual AHI was device-derived and may be influenced by device-specific event-detection algorithms [8]; device models were not uniform across the cohort, and changes in prescribed CPAP, leak data, and separate indices for residual obstructive and central events were not systematically available, limiting further characterization of elevated residual AHI values. Residual sleepiness was clinically assessed rather than defined exclusively by a standardized ESS threshold and was based on routine physician assessment without a predefined standardized classification algorithm. Moreover, baseline patient characteristics and comorbid conditions that may influence the burden or perception of daytime sleepiness, including obesity-related factors, expiratory airflow limitation, and cancer history, were not systematically incorporated into the classification of residual sleepiness [26,27,28]. Therefore, the symptomatic domain should not be interpreted as specifically reflecting residual OSA-related sleepiness. Perceived treatment benefit was assessed using a single binary question addressing overall perceived benefit and was not measured using a validated patient-reported outcome instrument or a domain-specific measure of symptoms, functioning, or quality of life. These non-standardized assessments may have introduced variability or misclassification in the symptomatic and patient-perceived treatment domains and may limit reproducibility across cohorts.

Finally, pairwise associations among treatment domains were exploratory, and nominal *p*-values were not adjusted for multiple comparisons; these findings should therefore be considered hypothesis-generating. The multidomain framework was intended to characterize heterogeneity across treatment outcomes rather than to define a composite measure of CPAP success. Simultaneously meeting all four predefined treatment-domain criteria should not be interpreted as a validated global treatment endpoint, as the individual domains may differ in clinical relevance and were not assigned predefined weights.

In conclusion, long-term CPAP management encompasses both continued engagement with care and multiple dimensions of treatment outcome. In this real-world cohort, approximately 70% of patients with sufficient follow-up opportunity attended the long-term assessment, while aggregate adherence prevalence was similar across follow-up assessments despite individual differences in adherence classification between observation intervals. Among patients with observed long-term follow-up data, CPAP adherence, device-derived respiratory control, residual symptoms, and perceived treatment benefit were only partially concordant, indicating that no single metric adequately captures long-term treatment status among assessed patients. Multidimensional reassessment may therefore be most useful for identifying discordant profiles requiring individualized interpretation rather than for defining a single measure of treatment success. Prospective studies should determine whether integrating such assessment into personalized follow-up pathways improves patient outcomes and healthcare delivery.

## Figures and Tables

**Table 1:** Baseline characteristics according to long-term follow-up attendance among patients with at least 30 months of potential follow-up (n = 114).

Characteristic	Overall Cohort (n = 114)	Did Not Attend T2 (n = 34)	Attended T2 (n = 80)	*p*-Value
Demographic and anthropometric characteristics				
Age, years	67.1 [55.8–74.7]	59.2 [50.1–72.7]	69.1 [59.3–75.0]	0.018
Male sex	95/114 (83.3%)	26/34 (76.5%)	69/80 (86.3%)	0.200
BMI, kg/m^2^	35.3 ± 7.2	36.6 ± 8.5	34.8 ± 6.6	0.279
Obesity	79/100 (79.0%)	24/31 (77.4%)	55/69 (79.7%)	0.795
Comorbidities				
Cardiovascular disease	95/113 (84.1%)	27/34 (79.4%)	68/79 (86.1%)	0.375
Diabetes mellitus	28/113 (24.8%)	11/34 (32.4%)	17/79 (21.5%)	0.221
Dyslipidemia	55/113 (48.7%)	15/34 (44.1%)	40/79 (50.6%)	0.525
Asthma	6/113 (5.3%)	2/34 (5.9%)	4/79 (5.1%)	1.000
COPD	18/113 (15.9%)	6/34 (17.6%)	12/79 (15.2%)	0.743
Renal disease	12/113 (10.6%)	3/34 (8.8%)	9/79 (11.4%)	1.000
History of malignancy	17/113 (15.0%)	5/34 (14.7%)	12/79 (15.2%)	0.947
Baseline sleep-related characteristics				
ESS score	7.5 [5–11]	7 [4.5–10]	8 [5–11]	0.750
Daytime sleepiness	58/87 (66.7%)	16/23 (69.6%)	42/64 (65.6%)	0.731
AHI, events/hour	39.9 [27.4–59.2]	44.0 [32.4–64.8]	39.0 [25.8–56.9]	0.175
ODI, events/hour	43.8 [28.8–64.6]	44.4 [37.1–69.2]	43.6 [27.5–64.4]	0.471
T_90_, %	26.1 [7.6–45.8]	30.0 [8.6–42.0]	24.2 [7.8–46.8]	0.860
Initial treatment characteristics				
Prescribed CPAP, cmH_2_O	8.0 [7.0–9.5]	7.5 [6.8–8.0]	8.0 [7.0–9.5]	0.131

Data are presented as mean ± standard deviation, median [interquartile range], or n/N (%), as appropriate. Continuous variables were compared using Student’s *t*-test when approximately normally distributed and the Mann–Whitney U test otherwise. Categorical variables were compared using the chi-square test or Fisher’s exact test, as appropriate. Analyses were restricted to patients with at least 30 months of potential follow-up from CPAP prescription to administrative database closure, independently of observed T2 attendance. Continuous-variable summaries were calculated using available cases. Available observations in the overall/did-not-attend/attended groups were: age, 114/34/80; BMI, 88/26/62; ESS, 70/19/51; AHI, 110/32/78; ODI, 67/22/45; T_90_, 90/27/63; and prescribed CPAP, 109/31/78. Abbreviations: BMI indicates body mass index; COPD indicates chronic obstructive pulmonary disease; ESS, Epworth Sleepiness Scale; AHI, apnea-hypopnoea index; ODI, oxygen desaturation index; T_90_, percentage of recording time spent with pulse oximetry oxygen saturation (SpO_2_) below 90%; CPAP, continuous positive airway pressure.

**Table 2:** Paired comparison of CPAP treatment outcomes at first and long-term follow-up.

Outcome	First Follow-Up (T1)	Long-Term Follow-Up (T2)	Paired n	Paired Difference (95% CI)	*p*-Value
Technical outcome					
Adherent, n/N (%)	74/94 (78.7)	75/94 (79.8)	94	+1.1 percentage points (−5.3 to +8.5)	1.000
Device-derived respiratory control					
Residual AHI, events/hour	5.0 [2.3–9.0]	5.1 [2.2–9.0]	85	+0.2 events/hour (−0.2 to +0.8)	0.222
Symptomatic outcome					
ESS score	3 [2–6]	3 [2–5]	68	0 points (−1 to 0)	0.368
Residual sleepiness, n/N (%)	11/85 (12.9)	11/85 (12.9)	85	0.0 percentage points (−8.2 to +8.2)	1.000
Patient-perceived outcome					
Perceived treatment benefit, n/N (%)	61/67 (91.0)	60/67 (89.6)	67	−1.5 percentage points (−9.0 to + 6.0)	1.000

Data are presented as median [interquartile range] or n/N (%), as appropriate, and refer exclusively to patients with the corresponding outcome available at both follow-up assessments. For continuous outcomes, paired differences are reported as median differences with 95% percentile bootstrap confidence intervals. For binary outcomes, paired differences are reported as absolute differences in proportions, expressed in percentage points, with 95% percentile bootstrap confidence intervals. Bootstrap resampling was performed at the patient level, preserving paired first follow-up/long-term follow-up observations, using 50,000 resamples. Wilcoxon signed-rank and McNemar tests were used for continuous and binary paired comparisons, respectively. Abbreviations: CPAP indicates continuous positive airway pressure; AHI, apnea-hypopnoea index; ESS, Epworth Sleepiness Scale.

**Table 3:** Multidomain assessment of CPAP treatment outcomes at the long-term follow-up.

Treatment Domain	Criterion Definition	Patients Meeting Criterion	Available Patients	Percentage (%)
Technical outcome	Device-derived CPAP adherence (≥4 h/night on ≥70% of nights)	75	94	79.8
Device-derived respiratory control	Residual AHI < 5 events/hour (predefined operational criterion) *	43	90	47.8
Symptomatic outcome	Absence of clinically assessed residual sleepiness	79	90	87.8
Patient-perceived outcome	Perceived benefit from CPAP treatment	67	75	89.3
Number of treatment-domain criteria met	≥1 domain criterion met	71	71	100
Number of treatment-domain criteria met	≥2 domain criteria met	67	71	94.4
Number of treatment-domain criteria met	≥3 domain criteria met	61	71	85.9
Number of treatment-domain criteria met	All 4 domain criteria met	32	71	45.1

Data are presented as n/N (%). For individual treatment domains, percentages were calculated using the number of patients with available data for each outcome as the denominator. The distribution of the number of treatment-domain criteria met was calculated among the 71 patients with complete data for all four treatment domains. The four domains were technical outcome (device-derived CPAP adherence), device-derived respiratory control (residual AHI < 5 events/hour), symptomatic outcome (absence of clinically assessed residual sleepiness), and patient-perceived outcome (perceived treatment benefit). * In a sensitivity analysis using residual AHI < 10 events/hour as an alternative operational criterion for device-derived respiratory control, 70/90 patients (77.8%) met the device-derived respiratory control criterion and 45/71 patients (63.4%) simultaneously met all four predefined treatment- domain criteria, compared with 43/90 (47.8%) and 32/71 (45.1%), respectively, using the primary threshold of <5 events/hour. Abbreviations: CPAP indicates continuous positive airway pressure; AHI, apnea-hypopnoea index.

**Table 4:** Factors associated with long-term follow-up attendance. Panel (A). Baseline factors associated with long-term follow-up attendance among patients with at least 30 months of potential follow-up (n = 114). Panel (B). Treatment characteristics at first follow-up associated with subsequent long-term follow-up attendance (T1 attendees with ≥30 months of potential follow-up, n = 102).

**Panel (A)**				
**Variable**	**Univariate OR (95% CI)**	***p*** **-Value**	**Parsimonious Adjusted Model OR (95% CI)**	***p*** **-Value**
Age, per 10-year increase	1.52 (1.06–2.18)	0.022	1.58 (1.10–2.28)	0.014
Male sex	1.93 (0.70–5.33)	0.205	2.35 (0.81–6.82)	0.115
BMI, per 5 kg/m^2^ increase	0.84 (0.61–1.15)	0.276	-	-
Baseline AHI, per 10 events/hour increase	0.89 (0.74–1.07)	0.222	-	-
Baseline T_90_, per 10-percentage-point increase	1.00 (0.84–1.18)	0.956	-	-
Baseline ESS, per 5-point increase	1.02 (0.57–1.82)	0.940	-	-
Cardiovascular disease	1.60 (0.56–4.57)	0.377	-	-
**Panel (B)**				
**Variable**	**Univariate OR (95% CI)**	***p*** **-Value**	**Age-Adjusted OR (95% CI)**	***p*** **-Value**
Adherent at T1	1.55 (0.48–4.96)	0.463	1.70 (0.51–5.67)	0.384
Residual AHI, per 5 events/hour increase	0.93 (0.62–1.38)	0.702	0.86 (0.57–1.28)	0.453
Residual sleepiness	0.57 (0.14–2.42)	0.448	0.62 (0.14–2.66)	0.517
Perceived treatment benefit	1.51 (0.27–8.38)	0.634	1.43 (0.24–8.41)	0.693

Panel A included patients with at least 30 months of potential follow-up from CPAP prescription to administrative database closure (n = 114; 80 attended T2 and 34 did not attend T2). Membership in the primary risk set was defined independently of observed T2 attendance. Univariate logistic regression models were fitted separately for age, sex, BMI, baseline AHI, baseline T_90_, baseline ESS score, and cardiovascular disease. To limit model complexity given the number of non-attendance events and missing baseline covariate data, the adjusted analysis was deliberately parsimonious and included age and sex only. Continuous predictors were scaled per 10-year increase in age, 5 kg/m^2^ increase in BMI, 10-events/hour increase in AHI, 10-percentage-point increase in T_90_, and 5-point increase in ESS score. The age- and sex-adjusted model included the entire primary T2 risk set (n = 114; 80 attended T2 and 34 did not attend T2), as age and sex were available for all patients. ORs >1 indicate a greater likelihood of attending T2. Panel B included patients who attended T1 and belonged to the primary T2 risk set, defined by at least 30 months of potential follow-up from CPAP prescription to administrative database closure (n = 102; 80 attended T2 and 22 did not attend T2). Each treatment characteristic measured at T1 was evaluated in a separate univariate logistic regression model and subsequently in a separate model adjusted for age. Models were based on available cases for the corresponding treatment characteristic and age. ORs > 1 indicate a greater likelihood of attending T2. Available cases for both the univariate and age-adjusted models were 99 for adherence, 93 for residual AHI, 88 for residual sleepiness, and 72 for perceived treatment benefit. *Abbreviations:* CPAP indicates continuous positive airway pressure; OR, odds ratio; CI, confidence interval; BMI indicates body mass index; AHI, apnea-hypopnoea index; T_90_, percentage of recording time spent with pulse oximetry oxygen saturation (SpO_2_) below 90%; ESS, Epworth Sleepiness Scale.

**Table 5:** Pairwise associations among CPAP treatment-outcome domains at the long-term follow-up.

Domain Pair	n	Phi Coefficient (ϕ)	Nominal Fisher’s Exact *p*-Value
Technical—Device-derived respiratory control	71	0.075	0.658
Technical—Symptomatic	71	0.076	0.460
Technical—Patient-perceived	71	0.278	0.073
Device-derived respiratory control—Symptomatic	71	0.114	0.459
Device-derived respiratory control—Patient-perceived	71	0.071	0.697
Symptomatic—Patient-perceived	71	0.330	0.027

Pairwise associations were evaluated among the 71 patients with complete data for all four treatment-outcome domains at long-term follow-up. Technical outcome was defined as device-derived CPAP adherence (≥4 h/night on ≥70% of nights), device-derived respiratory control as residual AHI < 5 events/hour, symptomatic outcome as absence of clinically assessed residual sleepiness, and patient-perceived outcome as the patient’s subjective report of benefit from CPAP treatment. The phi coefficient (ϕ) was used to quantify the strength of association between binary domains. Fisher’s exact test was used for all pairwise comparisons because of small expected cell counts. These analyses were exploratory, and nominal *p*-values were not adjusted for multiple comparisons. Phi coefficients were interpreted as measures of association within this cohort and not as evidence of independence between treatment-outcome domains. Abbreviations: CPAP indicates continuous positive airway pressure.

## Data Availability

The datasets used and/or analyzed during the current study are available from the corresponding author on reasonable request.

## Supplementary Materials

The following supporting information can be downloaded at: https://www.mdpi.com/article/10.3390/jcm15218296/s1. Table S1, Data availability and completeness across study assessments.; Table S2, Characteristics of patients with complete versus incomplete multidomain outcome data at long-term follow-up.; Table S3, Sensitivity analysis of long-term treatment outcomes restricted to T2 attendees belonging to the primary ≥ 30-month risk set.; Table S4, Sensitivity analyses of long-term follow-up attendance according to alternative potential follow-up thresholds and additional adjustment for potential follow-up duration and calendar year.; Figure S1, Schematic definition of primary follow-up risk sets according to potential follow-up duration and administrative database closure.
