# Supplementary material for: From CPAP Prescription to Long-Term Follow-Up: Care Pathway, Adherence, and Multidomain Treatment Outcomes in Obstructive Sleep Apnea
Source: J Clin Med. 2026 Oct 24;15(21):8296. doi: 10.3390/jcm15218296 (PMC13764190; doi:10.3390/jcm15218296)
Supplement: Supplementary file 1 [file jcm-15-08296-s001.zip › Supplementary Tables R1_clear.pdf]

Supplementary Table S1. Data availability and completeness across study assessments.

Variable	Baseline, n/N (%)	First follow-up (T1), n/N (%)	Long-term follow-up (T2), n/N (%)	Paired T1-T2, n
Primary follow-up risk set	-	147/172 (85.5)	114/172 (66.3)	-
Attendance within primary risk set	-	132/147 (89.8)	80/114 (70.2)	-
Demographic and anthropometric variables				
Age	172/172 (100)	-	-	-
Sex	172/172 (100)	-	-	-
BMI	132/172 (76.7)	-	-	-
Baseline OSA-related variables				
AHI	167/172 (97.1)	-	-	-
ODI	105/172 (61.0)	-	-	-
T_{90}	143/172 (83.1)	-	-	-
ESS score	115/172 (66.9)	99/136 (72.8)	81/97 (83.5)	68
Comorbidity				
Cardiovascular disease	171/172 (99.4)	-	-	-
Treatment-outcome variables				
CPAP adherence	-	132/136 (97.1)	94/97 (96.9)	94
Residual AHI	-	126/136 (92.6)	90/97 (92.8)	85
Residual sleepiness	-	120/136 (88.2)	90/97 (92.8)	85
Perceived treatment benefit	-	100/136 (73.5)	75/97 (77.3)	67

Data availability is reported as n/N (%). The first two rows report membership in the primary follow-up risk sets and attendance within those risk sets. Primary risk-set membership was determined independently of observed attendance according to potential follow-up duration at administrative database closure: ≥12 months for T1 and ≥30 months for T2. For baseline variables, N represents the 172 patients entering the CPAP care pathway. For treatment-outcome variables at T1 and T2, N represents all patients who actually underwent the corresponding assessment (136 and 97 patients, respectively), including assessments occurring outside the corresponding primary risk set. The paired T1-T2 column reports the number of patients with the corresponding variable available at both follow-up assessments and therefore included in paired longitudinal analyses. Missing measurements among patients attending a follow-up were considered item-level missing data and were distinguished from non-attendance. For perceived treatment benefit, missing data reflected absence of a documented assessment in the clinical record and were not classified as absence of perceived benefit.
Abbreviations: BMI indicates body mass index; AHI, apnea-hypopnea index; ODI, oxygen desaturation index; T_{90}, percentage of recording time spent with pulse oximetry oxygen saturation (SpO_2) below 90%; ESS, Epworth Sleepiness Scale; CPAP, continuous positive airway pressure.

Supplementary Table S2. Characteristics of patients with complete versus incomplete multidomain outcome data at long-term follow-up.

Characteristic	Complete multidomain data (n=71)	Incomplete multidomain data (n=26)	Descriptive p-value
Age, years	67.2 [58.3-74.4]	73.4 [66.3-75.2]	0.054
Male sex	64/71 (90.1%)	20/26 (76.9%)	0.103
BMI, kg/m^2	34.9 ± 6.8, n=55	31.7 ± 5.7, n=22	0.036
Baseline AHI, events/h	39.0 [27.2-57.1], n=70	28.0 [21.0-52.0], n=25	0.139
Baseline ESS	8 [5-11], n=49	7.5 [3.3-10.8], n=14	0.388

Adherent at T1	63/71 (88.7%)	13/26 (50.0%)	<0.001

The analysis included the 97 patients who underwent T2. Complete multidomain data were defined as concurrent availability of CPAP adherence, residual AHI, residual sleepiness, and perceived treatment benefit at T2. Comparisons were descriptive and intended to characterize potential selection into the complete-case multidomain cohort rather than to identify predictors of data completeness. Continuous variables are presented as mean ± standard deviation or median [interquartile range], as appropriate, and categorical variables as n/N (%). Available-case denominators are reported where data were missing. P-values are unadjusted and should be interpreted descriptively.
Abbreviations: BMI indicates body mass index; AHI, apnea-hypopnea index; ODI, oxygen desaturation index; ESS, Epworth Sleepiness Scale; T1, first follow-up assessment.

Supplementary Table S3. Sensitivity analysis of long-term treatment outcomes restricted to T2 attendees belonging to the primary ≥30-month risk set.

Outcome	All observed T2 assessments	T2 attendees in primary ≥30-month risk set
Cohort size	97	80

CPAP adherence	75/94 (79.8%)	62/77 (80.5%)
Residual AHI <5 events/hour	43/90 (47.8%)	34/75 (45.3%)
Residual AHI <10 events/hour	70/90 (77.8%)	59/75 (78.7%)
No residual sleepiness	79/90 (87.8%)	63/74 (85.1%)
Perceived treatment benefit	67/75 (89.3%)	53/61 (86.9%)
Complete four-domain data	71/97 (73.2%)	58/80 (72.5%)
All four criteria, AHI <5	32/71 (45.1%)	24/58 (41.4%)
All four criteria, AHI <10	45/71 (63.4%)	36/58 (62.1%)

Data are presented as n/N (%). The restricted sensitivity cohort included only T2 attendees belonging to the primary ≥30-month risk set. Available-case denominators were used for individual domains, whereas the proportion simultaneously meeting all four predefined criteria was assessed among patients with complete four-domain data. Residual AHI <5 events/hour was the primary criterion for respiratory control; <10 events/hour was examined as a sensitivity definition. No formal statistical comparison between the full observed T2 cohort and the restricted sensitivity cohort was performed because the latter represents a nested subset of the former; the analysis was intended to assess the robustness of treatment-outcome estimates descriptively.

Abbreviations: CPAP indicates continuous positive airway pressure; AHI, apnea-hypopnea index; T2, long-term follow-up assessment.

Supplementary Table S4. Sensitivity analyses of long-term follow-up attendance according to alternative potential follow-up thresholds and additional adjustment for potential follow-up duration and calendar year.

Panel A. Alternative minimum potential follow-up thresholds defining the T2 risk set.

Minimum potential follow-up	Risk set	Attendance	Age OR/10 y (95% CI)	p-value	Age + sex aOR/10 y (95% CI)	p-value
≥24 months	130	89/130 (68.5%)	1.55 (1.11-2.15)	0.010	1.60 (1.14-2.24)	0.007
≥30 months (primary)	114	80/114 (70.2%)	1.52 (1.06-2.18)	0.022	1.58 (1.10-2.28)	0.014
≥36 months	92	67/92 (72.8%)	1.33 (0.88-2.00)	0.172	1.39 (0.91-2.11)	0.124

Panel B. Additional adjustment for potential follow-up duration in the primary ≥30-month risk set.

Variable	Adjusted OR (95% CI)	p-value
Age, per 10-year increase	1.58 (1.10-2.29)	0.014
Male sex	2.35 (0.81-6.83)	0.115
Potential follow-up, per 1-year increase	1.00 (0.63-1.57)	0.995

Panel C. Additional adjustment for calendar year of CPAP prescription in the primary ≥30-month risk set.

Prescription year	Primary T2 risk set, n	Attended T2, n	Attendance, %
2019	1	0	0.0

2020	2	1	50.0
2021	27	19	70.4
2022	38	29	76.3
2023	46	31	67.4

Variable	Adjusted OR (95% CI)	p-value
Age, per 10-year increase	1.59 (1.10-2.31)	0.013
Male sex	2.38 (0.82-6.91)	0.112
Calendar year of CPAP prescription, per 1-year increase	1.14 (0.72-1.82)	0.577

Panel A evaluated the sensitivity of long-term follow-up attendance to alternative minimum potential follow-up thresholds used to define the T2 risk set. Risk-set membership was determined exclusively by potential follow-up duration from CPAP prescription to the administrative database closure date (June 30, 2026) and was independent of observed T2 attendance. The primary analysis used a minimum potential follow-up of 30 months; thresholds of 24 and 36 months were examined as sensitivity analyses. Odds ratios for age are expressed per 10-year increase. Adjusted models included age and sex.

Panel B evaluated whether the association between age and T2 attendance in the primary ≥30-month risk set was influenced by differences in potential follow-up duration. The model included the entire primary T2 risk set (n=114). Potential follow-up duration was expressed per 1-year increase. ORs >1 indicate a greater likelihood of attending T2.

Panel C evaluated whether the association between age and T2 attendance was influenced by calendar time. The model included age, sex, and calendar year of CPAP prescription and included the entire primary T2 risk set (n=114). Calendar year was modeled as a continuous variable and expressed per 1-year increase. Year-specific attendance estimates were descriptive. Formal comparisons across individual years were not performed because of the very small numbers of patients prescribed CPAP in 2019 and 2020.

Abbreviations: OR indicates odds ratio; CI, confidence interval; T2, long-term follow-up assessment; CPAP, continuous positive airway pressure.

SUPPLEMENTARY FIGURES LEGEND

Supplementary Figure S1. Schematic definition of primary follow-up risk sets according to potential follow-up duration and administrative database closure.

Schematic representation of the approach used to define the primary follow-up risk sets according to time since CPAP prescription and the administrative database closure date (June 30, 2026). Risk-set membership was determined independently of observed attendance. The primary T1 risk set included patients with at least 12 months of potential follow-up from CPAP prescription to database closure, whereas the primary T2 risk set included patients with at least 30 months of potential follow-up. The 30-month threshold represented a pragmatic operational definition intended to provide sufficient opportunity for the clinically scheduled approximately three-year assessment rather than to define a fixed assessment window. The positions of T1 and T2 in the schematic patient examples are illustrative and do not represent predefined visit windows or exact assessment timing. Patients below the corresponding threshold were excluded from the primary attendance denominator irrespective of whether an early assessment had already occurred; observed assessments outside the corresponding primary risk set could nevertheless contribute to treatment-outcome analyses. Alternative minimum potential follow-up thresholds of 24 and 36 months were examined in sensitivity analyses. The hypothetical examples illustrate a patient included in both primary risk sets who attended both assessments (A), a patient with sufficient potential follow-up who did not attend T2 (B), a patient with insufficient potential follow-up for inclusion in the primary T2 risk set (C), and a patient who underwent an early T1 assessment but remained outside the primary T1 risk set because potential follow-up was <12 months (D).

Abbreviation: CPAP, continuous positive airway pressure; T1, first follow-up assessment; T2, long-term follow-up assessment.
